# Impact of physiological ionic strength and crowding on kinesin-1 motility

**DOI:** 10.1247/csf.24074

**Published:** 2025-01-08

**Authors:** Misaki Sagawa, Kazuhiro Oiwa, Hiroaki Kojima, Ken’ya Furuta, Keitaro Shibata

**Affiliations:** 1 Graduate School of Life Science, University of Hyogo, Harima Science Park City, Hyogo 678-1297, Japan; 2 Advanced ICT Research Institute, National Institute of Information and Communications Technology, Kobe, Hyogo 651-2492, Japan; 3 Department of Cell Biology, Graduate School of Medical Sciences, Tokushima University, Tokushima, Tokushima 770-8503, Japan

**Keywords:** kinesin motility, molecular crowding, ionic strength, intracellular transport, processivity of molecular motors

## Abstract

The motility of biological molecular motors has typically been analyzed by in vitro reconstitution systems using motors isolated and purified from organs or expressed in cultured cells. The behavior of biomolecular motors within cells has frequently been reported to be inconsistent with that observed in reconstituted systems in vitro. Although this discrepancy has been attributed to differences in ionic strength and intracellular crowding, understanding how such parameters affect the motility of motors remains challenging. In this report, we investigated the impact of intracellular crowding in vitro on the mechanical properties of kinesin under a high ionic strength that is comparable to the cytoplasm. Initially, we characterized viscosity in a cell by using a kinesin motor lacking the cargo-binding domain. We then used polyethylene glycol to create a viscous environment in vitro comparable to the intracellular environment. Our results showed that kinesin frequently dissociated from microtubules under high ionic strength conditions. However, under conditions of both high ionic strength and crowding with polymers, the processive movement of kinesin persisted and increased in frequency. This setting reproduces the significant variations in the mechanical properties of motors measured in the intracellular environment and suggests a mechanism whereby kinesin maintains motility under the high ionic strengths found in cells.

## Introduction

Protein motors are essential ATPases that play a critical role in intracellular transport (Vale *et al.*, 1985; Vale and Oosawa, 1990). The continuing development of in vitro reconstitution systems has facilitated our understanding of the physical and chemical properties of these motors (Block *et al.*, 1990; Howard *et al.*, 1989; Vale *et al.*, 1996; Vale and Toyoshima, 1988). These systems, when combined with single-molecule measurement techniques, have enabled detailed characterization of motor functions, such as force generation and step size (Svoboda *et al.*, 1993; Yildiz *et al.*, 2004).

Simplifications are often made for in vitro studies to meet experimental requirements and allow precise two-dimensional measurements. Motors are typically bound to glass substrates or polystyrene microbeads, allowing detailed analysis of their interactions with cytoskeleton filaments, which act as tracks for motors. For example, kinesin has been shown to move along microtubules with a step size of 8 nm exhibiting highly processive movement (Isojima *et al.*, 2016; Svoboda *et al.*, 1993; Yildiz *et al.*, 2004).

However, the intracellular environment where these motors operate vastly differs from the controlled conditions of in vitro reconstituted systems. Factors such as ionic strength, pH and high-molecular-weight polymers in molecular crowding, which strongly affect motor activity, greatly alter the distribution of motors locally in cells (Debold *et al.*, 2008; Luby-Phelps, 2013; Ross, 2016; Sabharwal and Koushika, 2019; Verhey *et al.*, 1998; Wang and Sheetz, 2000). Additionally, post-translational modifications (e.g., phosphorylation) and regulation of activity through interactions with regulatory proteins further influence motor behavior (Banerjee and Gunawardena, 2023; Totsukawa *et al.*, 1999). For example, kinesin-driven vesicle transport in cells (velocity: 0.8–4.0 μm/s) is notably faster than kinesin motility in vitro (velocity: 0.6–0.8 μm/s), a discrepancy attributed to the oversimplified conditions in vitro (Hill *et al.*, 2004; Hirschberg *et al.*, 1998; Kural *et al.*, 2005; Naoi *et al.*, 2024; Ross, 2016). In particular, the ionic strength used in most in vitro reconstituted systems is much lower than in cells. Ionic strength of the solution as high as that of the cytoplasm reduces electrostatic forces, weakening motor-cytoskeleton interactions and significantly reducing motor processivity. Furthermore, the intracellular environment is far more crowded compared to reconstitution systems. The environment contains macromolecules at concentrations of 80–400 mg/mL, creating significant physical constraints (Zimmerman and Trach, 1991; Rivas *et al.*, 2004). Previous study has shown that small molecular crowding agents can impede kinesin-1 motility by interfering with motor domain diffusion (Sozański, 2015). In cells, motors achieve highly regulated and efficient intracellular trafficking despite these physical constraints of high ionic strength and molecular crowding, however, the mechanisms that make this possible remain unresolved.

In this study, we focused on kinesin-1, a microtubule motor protein. We compared the motility and diffusivity of kinesin-1 between intracellular and in vitro environments and searched for factors that enable kinesin-1 to move processively within cells. We found that polymers that could mimic intracellular molecular crowding enhanced the processivity of kinesin at a high ionic strength. This finding raises the possibility that the presence of polymers and other substances in a crowded intracellular environment can significantly affect the motility of molecular motors.

## Materials and Methods

### Plasmids

K430-3 × SNAP was constructed using a modified pCold^TM^ ProS2 DNA vector (Takara Bio Inc.: 3371; Shiga, Japan), where the EcoRI site in the multi-cloning site was specifically engineered to be positioned immediately downstream of the HRV 3C protease recognition sequence. The sequence encoding amino acids 1–430 of rat kinesin-1 followed by three tandem SNAP-tag sequences and a FLAG-tag was inserted between EcoRI and HindIII sites using Gibson assembly. GS linkers were incorporated between each genetic element. The inserted gene fragments were generated by PCR using plasmids from a previous study as templates (Shibata *et al.*, 2012). For intracellular microtubule visualization, GFP-tubulin was constructed by introducing a 15-amino acid linker sequence between GFP and tubulin in EGFP-tubulin-6 (addgene: #56450; Watertown, MA, USA). Venus-2 × FYVE was generated by replacing mCherry in pmCherry-2′FYVE (addgene: #140050). Dendra2 (DDR2)-K was created by substituting amino acids 1–327 of kinesin in pCold ProS2/K430-3 × SNAP with DDR2 using Gibson assembly. The plasmid for DDR2-K expression in mouse embryonic fibroblast (MEF) cells was constructed by replacing mCherry in pmCherry-C1 with DDR2-K using Gibson assembly.

### Mammalian cell culturing and expression

MEF cells were cultured in DMEM (High Glucose) (Nacalai Tesque, Inc.: 08458-45; Kyoto, Japan) supplemented with 10% FBS, 1 × MEM nonessential amino acids solution (Nacalai Tesque, Inc.: 06344-56) and 10 μg/μL penicillin-streptomycin. The cells were maintained at 37°C in a 5% CO_2_ environment. Electroporation was performed with NEPA21 TypeII (Nepagene, Chiba, Japan) according to the manufacturer’s instructions. The cells were cultured overnight.

### Protein purification

In principle, the recombinant proteins were purified as described previously (Shibata *et al.*, 2012). A brief description, including some modifications, is given below. Recombinant proteins were expressed in *E. coli* BL21 (DE3) cells. Protein expression was induced with 0.1 mM IPTG (Fujifilm Wako Pure Chemicals Corporation: 099-02534; Osaka, Japan) for 24 h at 15°C. Cells were harvested, sonicated and the soluble fraction collected following ultracentrifugation. His-tag fusion proteins were purified by nickel ion-immobilized metal ion affinity chromatography (Profinity^TM^ IMAC Resin, Ni-charged, Bio-Rad: #156-0133; Hercules, CA, USA) and eluted with an elution buffer containing 0.3 M imidazole. HRV 3C protease (Takara Bio Inc.: #7360) was used to cleave the His-tag and a solubilizing tag, ProS2. As the FLAG tag was fused to the C-terminus of target proteins, these proteins were purified by affinity chromatography using an anti-FLAG^®^M2 antibody affinity agarose gel and eluted with an elution buffer containing 3× FLAG peptide (Sigma-Aldrich: F4799-4MG; St. Louis, MO, USA). For fluorescent labeling, K430-3 × SNAP and SNAP-Surface^®^ Alexa Fluor^®^ 647 (New England Biolabs: S9136S; Ipswich, MA, USA) were mixed 1:10 and incubated at room temperature for 60 min. The buffer was replaced with BRB80 buffer (80 mM PIPES-KOH, 2 mM MgCl_2_, 1 mM EGTA, pH 6.8) containing 0.5 mM dithiothreitol using a buffer displacement column, Nap5, (Cytiva: 17085301; MA, USA) to remove unreacted SNAP-surface Alexa Fluor 647.

### Preparation of tubulin and microtubules

Tubulins were purified from porcine brain using a high-molarity PIPES buffer (1 M PIPES, 20 mM EGTA, and 10 mM MgCl_2_; pH adjusted to 6.8 using KOH) as described previously (Castoldi and Popov, 2003). Tubulin was labeled with ATTO 550 NHS-ester (ATTO-TEC, AD 550-31). ATTO550 labeled microtubules were polymerized by copolymerizing the ATTO550-tubulin (labeling stoichiometry of 25.5% for ATTO550) and unlabeled tubulin at a ratio of 1:2.2 for 30 min at 37°C, and stabilized with 40 μM paclitaxel (Sigma-Aldrich: T1912).

### Protein introduction

Fuse-It-P (Ibidi GmbH, Gräfelfing, Germany) reagent was mixed with 40 μL of 100 μg/mL Alexa647 labeled K430. The solution was vortexed overnight to ensure complete mixing and sonicated in ice-cold water for 10 min. The volume of the Fuse-it solution was increased by adding 55 μL of 20 mM HEPES-NaOH (pH 7.4) to give an approximate final volume of 100 μL. This solution was vortexed for 30 s. A small amount of the Fuse-it solution (10–25 μL) was diluted in 500 μL PBS and vortexed for 30 s. The culture medium was replaced with the diluted Fuse-it solution. Cells were incubated for 5 min at 37°C. After incubation, the Fuse-it solution was replaced with fresh DMEM to terminate the fusion process.

### Buffer for imaging

For live cell imaging, cells were maintained in Live Cell Imaging (LCI) solution (20 mM HEPES-NaOH, 140 mM NaCl, 2.5 mM KCl, 1.8 mM CaCl_2_, 1.0 mM MgCl_2_, pH 7.4). For the reconstitution system, intracellular-like (IC) buffer (281 mM HEPES-KOH, 47.5 mM K_2_HPO_4_, 2.5 mM Na_2_HPO_4_, 10 mM NaHCO_3_, 13 mM MgSO_4_, 0.5 mM MgCl_2_, pH 7.0, an ionic strength 273.5 mM) was prepared based on previously reported intracellular ion compositions (Andersen, 2013; Ozawa and Fukuda, 2014). The control observations were performed using BRB12 buffer (12 mM PIPES-KOH pH 6.8, 2 mM MgCl_2_, 1 mM EGTA-KOH , ionic strength 25 mM), which has been conventionally used for single molecule measurements. Physiological viscosity was reproduced by the addition of polyethylene glycol of different molecular weights (average molecular weights 1000, 8000, and 20000, referred to as PEG1k, PEG8k, and PEG20k, respectively). The ionic strength of buffers was determined using the Debye-Hückel equation


$$I=\frac{1}{2}\sum_{i}c_{i}{z_{i}}^{2}$$ (1)

where ci is the molar concentration (in mol/L), zi is the charge number.

### Fluorescence microscopy

Live cell imaging and Photo-converted Intensity Profile Expansion (PIPE) method were performed using a confocal microscope LSM 880 with Airyscan (Zeiss, Oberkochen, Germany). Images were acquired with an oil immersion objective lens (Alpha Plan-Apochromat 100×/1.46 OilDIC M27 Elyra, Zeiss) in the Airyscan Fast mode. Motility assays were performed using a homebuilt total internal reflection fluorescence microscope based on a Ti-E microscope (Nikon Eclipse) equipped with an oil immersion objective lens (CFI Apochromat TIRF 60 × C Oil, NA/1.49) and a motorized stage (H117E1N4, Prior), as described previously (Ibusuki *et al.*, 2022).

### Imaging

MEF cells were seeded onto 35 mm glass base dishes (IWAKI, Tokyo, Japan) and cultured overnight for live cell imaging. The culture medium was replaced with the LCI solution. Live cell imaging was performed using the laser scanning confocal microscope system LSM880 Airyscan (Zeiss) at 25°C. Images were acquired at 488 and 633 nm excitation wavelengths with emission collected through a BP 495–500 + LP 570 filter. Time-lapse images were captured every 0.2 s for a total duration of 2000 frames. The pixel size was 60 × 60 nm.

For PIPE in BRB12 and IC buffer, flow chambers were constructed using two cover glasses of different sizes (24 × 32 mm non-treated, custom-made; 18 × 18 mm C218181; Matsunami Glass Ind., Ltd., Osaka, Japan) with Parafilm spacers (Heathrow Scientific LLC, Vernon Hills, IL, USA). The surface of 24 × 32 cover glass was coated with DDS to prevent the non-specific binding of proteins. Details of the coating process have been described previously (Helenius *et al.*, 2006). The internal size of the chamber was 18 mm long, 2.5 mm wide and 150 μm high. The flow chamber was filled with 10% v/v Alpha Tubulin Monoclonal antibody (66031-1-Ig, Proteintech, San Diego, CA, USA) in BRB12 buffer and incubated for 5 min at room temperature. The flow chamber was then filled with 1% w/v Pluronic F127 in BRB12 buffer and incubated for 5 min at room temperature. The flow chamber was filled with ~0.7 mg/mL casein (Nacalai Tesque: 07319-82) and 20 μM paclitaxel in BRB12 buffer and incubated for 5 min at room temperature. The culture medium was exchanged with LCI solution for PIPE in MEF cells. All measurements were performed using the LSM880 Airyscan. Excitation was performed using 488 nm (5%–10% power) and 561 nm (70% power) lasers. Photoactivation was achieved using 405 and 488 nm (100% power) at 2 × 2 pixels with 5000 iterations. The field of view was set to 288 × 288 pixels with a zoom factor of 1.8×. For each measurement, the acquisition sequence consisted of 10 pre-activation frames followed by 500 post-activation frames.

Flow chambers were constructed using two cover glasses of different sizes (24 × 32 mm; 18 × 18 mm) with Parafilm spacers for the motility assay in the in vitro reconstitution system. The surface of 24 × 32 cover glass was coated with DDS. The flow chamber was filled with 10% v/v Alpha Tubulin Monoclonal antibody in BRB12 buffer and incubated for 5 min at room temperature. The flow chamber was then filled with 1% w/v Pluronic F127 in BRB12 buffer and incubated for 5 min at room temperature. The flow chamber was filled with ~0.7 mg/mL casein and 20 μM paclitaxel in BRB12 buffer and incubated for 5 min at room temperature. The flow chamber was then filled with ATTO 550 labeled microtubules and 20 μM paclitaxel in BRB12 buffer and incubated for 5 min at room temperature. After washing with 20 μL of IC buffer, the flow chamber was filled with 15 μL of the final solution [IC or BRB12 buffer with 0%–21% PEG1k, PEG8k, and PEG20k, 0.218 mg/mL glucose oxidase, 0.04 mg/mL catalase, 2 mM dithiothreitol, 1 mM ATP, 10 μM paclitaxel, 0.7 mg/mL casein and 25 mM glucose].

### Viscosity measurements

The diffusion coefficient was determined using the PIPE method described in previous studies (Gura Sadovsky *et al.*, 2017). The Stokes radius of DDR-K was calculated using the Stokes-Einstein equation (1) from the diffusion coefficient of DDR-K in BRB80 determined by the PIPE method and the viscosity of BRB80 approximated by the viscosity of water at 25°C (0.89 mPa·s). The intracellular viscosity was calculated using the equation (1) from the diffusion coefficient of DDR-K in cells determined by the PIPE method and the Stokes radius of DDR-K determined above.


$$D=\;\frac{k_{B}T}{6\pi \eta r}$$ (2)

*D* is the diffusion coefficient, *k*_B_ is the Boltzmann constant, *T* is the absolute temperature, *η* is the viscosity of the medium and *r* is the Stokes radius of the particle.

### Data analysis

The velocity and run length of each K430 were analyzed using standalone custom software (Mark 2.6). The position of the K430 was determined using a two-dimensional Gaussian fitting algorithm in Mark 2.6. The velocity was calculated by linear least-squares fitting to each trace. The analysis included fluorescent spots that moved unidirectionally for more than 400 nm for single-molecule motility assays in MEF. For single-molecule motility assays in the reconstitution system, fluorescent spots that dissociate from the microtubule in less than 200 ms were excluded from the analysis. The run length was measured from the start of their movement until they dissociated from microtubules. The mean value of the run length was determined by nonlinear fitting of the cumulative fraction to a one-phase exponential decay model and corrected by following a previously established method (Thompson *et al.*, 2013). The processive run frequency (frequency of K430 molecules remaining on microtubules for over 200 ms) was calculated by counting the number of binding K430 to microtubules per unit of length and time (μm^–1^ · s^–1^).

## Results

### Motility of recombinant kinesin in cells

K430, a rat kinesin-1 construct containing amino acids 1–430, was used. Despite lacking the tail domain involved in cargo binding and interaction with accessory proteins, K430 functions as a minimal unit necessary for processive motility (Shibata *et al.*, 2012). The movement of recombinant K430 introduced into cells was analyzed to test the hypothesis that unknown cellular factors promote kinesin-1 motility.

For single-molecule detection in cells, a recombinant K430 protein with three C-terminal SNAP tags was constructed and labeled with benzylguanine-Alexa647 because of the requirement for strong fluorescent labeling. Fluorescence intensity analysis confirmed an average of 2.6 Alexa647 molecules per K430 homodimer. The fluorescently labeled K430 was encapsulated in liposomes and introduced into MEF cells through membrane fusion.

**Fig. 1:**
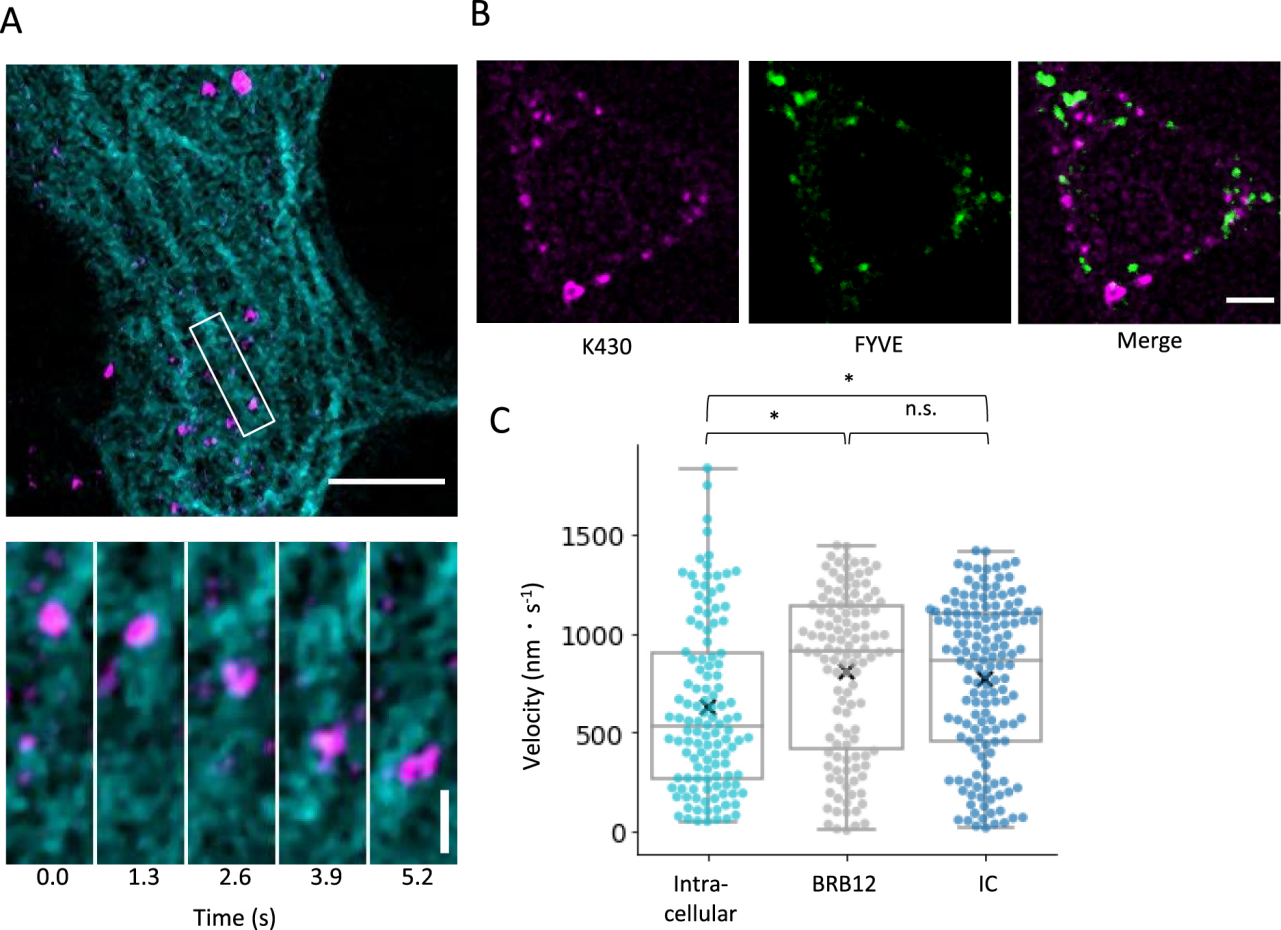
Single-molecule analysis of K430 motility in living cells (A) Single-molecule imaging of K430 in living cells. Cyan: transiently expressed tubulin-GFP; magenta: K430 introduced via liposome-based delivery. The upper panel shows a general view. Scale bar represents 5 μm. The lower sequence of images is a magnified view of the white square in the upper panel, with images acquired every 1.3 s. K430 is moving on a microtubule towards the bottom of the images. Scale bar denotes 1 μm. (B) Live cell imaging of K430 and FYVE, an early endosome marker. Magenta: liposome-delivered K430; green: transiently expressed 2× FYVE-Venus. Co-localization of K430 and FYVE cannot be confirmed. Scale bar represents 5 μm. (C) Comparison of K430 velocities in cells and BRB12 and IC buffers. The arithmetic mean ± SD velocity values were 622 ± 429 nm/s, *n* = 121, N = 14 in cells; 803 ± 414 nm/s, *n* = 122, N = 3 in BRB12 buffer; and 770 ± 400 nm/s, *n* = 150, N = 3 in IC buffer. The test was performed using the Dunnett method. *p<0.05.

Live-cell imaging at 25°C revealed numerous fluorescent puncta moving directionally along microtubules (Fig. 1A and Movie S1). Co-localization analysis with an early endosome marker (the FYVE domain fused with Venus in tandem) (Gillooly *et al.*, 2000) confirmed that K430 movement was not associated with endosomal vesicles (Fig. 1B, S1 and Movie S2), indicating puncta movement was driven by direct walking of K430 on intracellular microtubules.

Quantitative analysis of K430 motility focused on puncta moving continuously over 0.4 μm in a consistent direction. For comparison, a single-molecule motility assay was also conducted in an in vitro reconstitution system using the conventional BRB12 buffer and an IC buffer with elevated ionic strength. The mean velocity in cells was slower and showed greater variability than under both buffer conditions (Fig. 1C). In fact, various movement patterns were observed in cells, including random and bidirectional movements, probably due to K430 diffusion and/or walking while changing scaffold microtubules. These erratic movements were excluded from this analysis to calculate the accurate velocity of processive movement.

Previous studies have reported that full-length kinesin-1 expressed in cultured cells moves at 0.78 ± 0.11 μm/s (Cai *et al.*, 2007); however, faster velocities of 1 μm/s in cultured cells at 37°C have also been reported (Kapitein *et al.*, 2010). Considering differences in the kinesin construct, cell types and/or experimental systems, our experimental results (0.62 ± 0.43 μm/s, mean ± SD) are close to the range of these velocities reported in the previous studies.

### Intracellular viscosity measurements using the PIPE method

The reduced mean velocity and increased variability of K430 motility in cells suggested the potential effects of molecular crowding on motor protein dynamics. K430 diffusion in cells and intracellular viscosity were measured using the PIPE method (Gura Sadovsky *et al.*, 2017) to quantitatively evaluate how intracellular crowding affects K430 mobility and influences its motile velocity.

**Fig. 2:**
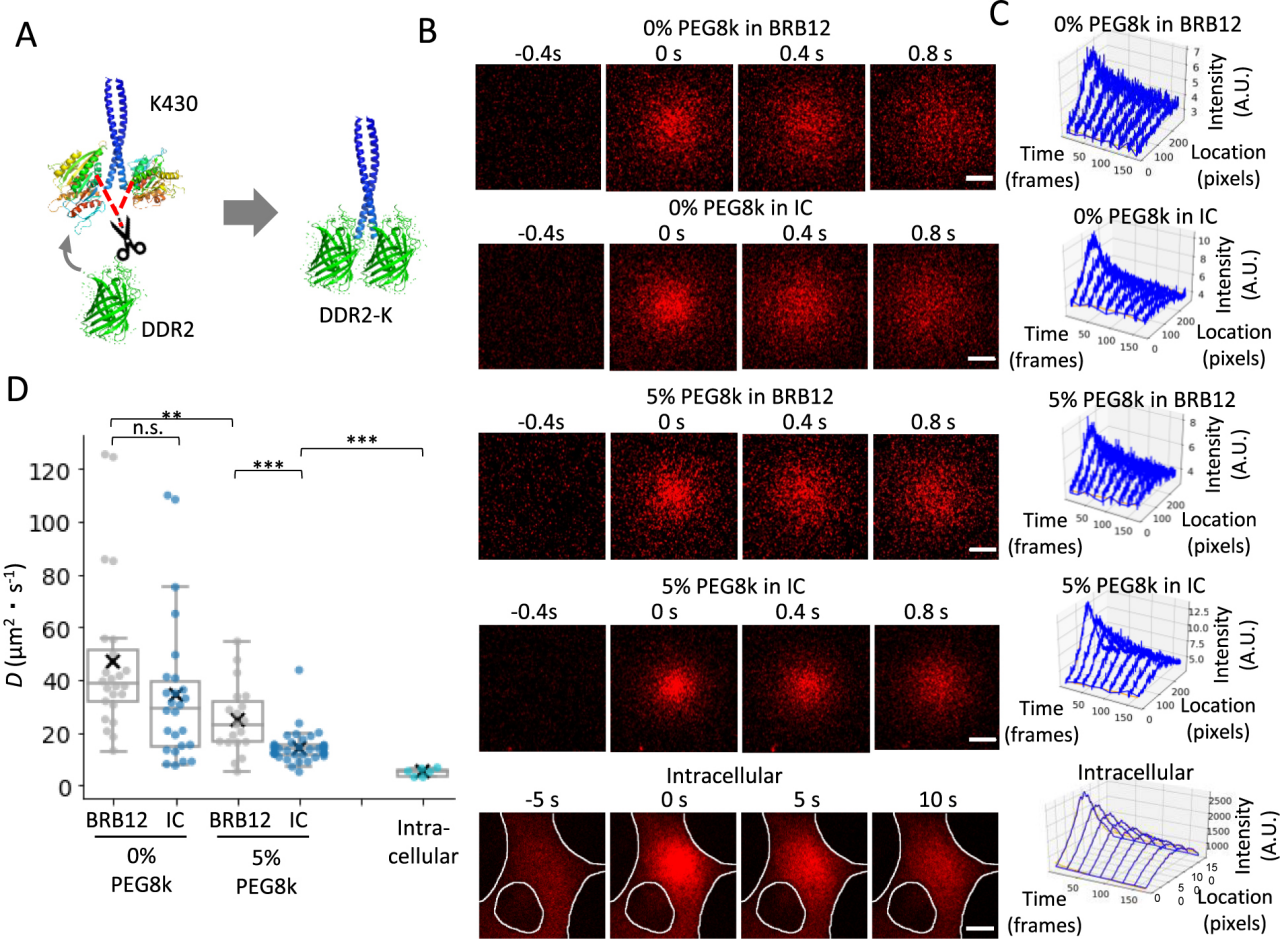
Viscosity measurements using the PIPE method (A) Schematic representation of DDR2-K. The K430 head domain (a.a. 1–327) was replaced by DDR2. Protein structures were obtained from the Protein Data Bank (PDB IDs: 1N6M and 2VZX). (B) Time-lapse images were taken before and after 405 nm pulse laser photoactivation of DDR2 in conditions, 0% PEG8k in BRB12 buffer, 0% PEG8k in IC buffer, 5% PEG8k in BRB12 buffer, 5% PEG8k in IC buffer and MEF cells. Red indicates photo-converted DDR2. The white lines indicate the outlines of cells and nuclei. Scale bars represent 5 μm. (C) Time series plots of fluorescence intensity distributions in conditions, 0% PEG8k in BRB12 buffer, 0% PEG8k in IC buffer, 5% PEG8k in BRB12 buffer, 5% PEG8k in IC buffer and MEF cells. Blue curves represent fluorescence intensity profiles, whereas orange curves show the fitting curves. (D) Diffusion coefficients of DDR2-K in conditions, 0% PEG8k in BRB12 buffer, 0% PEG8k in IC buffer, 5% PEG8k in BRB12 buffer, 5% PEG8k in IC buffer and MEF cells. The mean ± SD values are 47.14 ± 29.65 μm^2^/s (*n* = 24, N = 3) for 0% PEG8k in BRB12 buffer, 34.44 ± 27.71 μm^2^/s (*n* = 26, N = 3) for 0% PEG8k in IC buffer, 24.85 ± 13.21 μm^2^/s (*n* = 19, N = 3) for 5% PEG8k in BRB12 buffer, 14.26 ± 6.08 μm^2^/s (*n* = 38, N = 3) for 5% PEG8k in IC buffer and 4.86 ± 1.55 μm^2^/s (*n* = 7, N = 6) for cells. **p<0.01. ***p<0.001.

Initially, a model molecule, DDR2-K, was engineered by replacing the motor domain of K430 with the photoconvertible fluorescent protein DDR2. Although this chimera protein lacks microtubule-binding capability, it is expected to maintain a size and shape comparable to K430, based on the crystal structure of the K430 motor domain and DDR2 (Fig. 2A). After 405 nm laser irradiation, photoconverted DDR2-K diffused and its distribution was expanded in BRB 12 and IC buffer (Fig. 2B and C). To investigate the effects of molecular crowding on diffusion, parallel experiments were performed in each buffer containing 5% PEG8k. From these expanding behaviors, the diffusion coefficients of DDR2-K were determined (Fig. 2D). The combination of high ionic strength and PEG significantly suppressed DDR2-K diffusion, which should be attributed to the influence of ions on the structure and hydration shell formation of DDR2-K and PEG in solution.

The diffusion coefficient of DDR2-K was subsequently measured in cells, yielding a value of 4.86 ± 0.63 μm^2^/s (mean ± SEM, *n* = 6) (Fig. 2 D). The calculation of intracellular viscosity required determination of the Stokes radius of DDR2-K in cells. Previous studies have shown that the Stokes radius of proteins is constant regardless of the concentration of crowder (Sozański, 2015), thus allowing the assumption that the intracellular Stokes radius of DDR2-K equals that in the IC buffer. Due to its similarity to cellular ionic composition and known viscosity, 0% PEG8k in IC buffer was selected for Stokes radius calculation of DDR2-K. Using the Stokes-Einstein equation with the diffusion coefficient (34.4 ± 12.3 μm^2^/s) and IC buffer viscosity (approximated to water viscosity at 25°C, 0.89 mPa·s), the intracellular Stokes radius of DDR2-K was calculated to be 7.1 ± 2.6 nm. The dimeric DDR2-K contains six SNAP tags at the tail domain, with each SNAP tag exhibiting an approximately globular form with a 7 nm diameter (PDB ID: 3KZY). In conjunction with previously reported kinesin structures (Sack *et al.*, 1997), this calculated Stokes radius for DDR2-K represents a structurally feasible value. Application of the Stokes-Einstein equation to this Stokes radius and the intracellular diffusion coefficient yielded a intracellular viscosity of 6.3 ± 2.5 mPa·s. The approximately ten-fold reduction in the diffusion coefficient between IC and cellular environments demonstrates that the intracellular environment significantly restricts the free diffusion of molecules with a size similar to K430, suggesting that this restricted mobility may be a crucial factor affecting the motility characteristics of K430.

### Molecular crowding effects enhance kinesin processivity in a physiological ionic strength and crowding condition

**Fig. 3:**
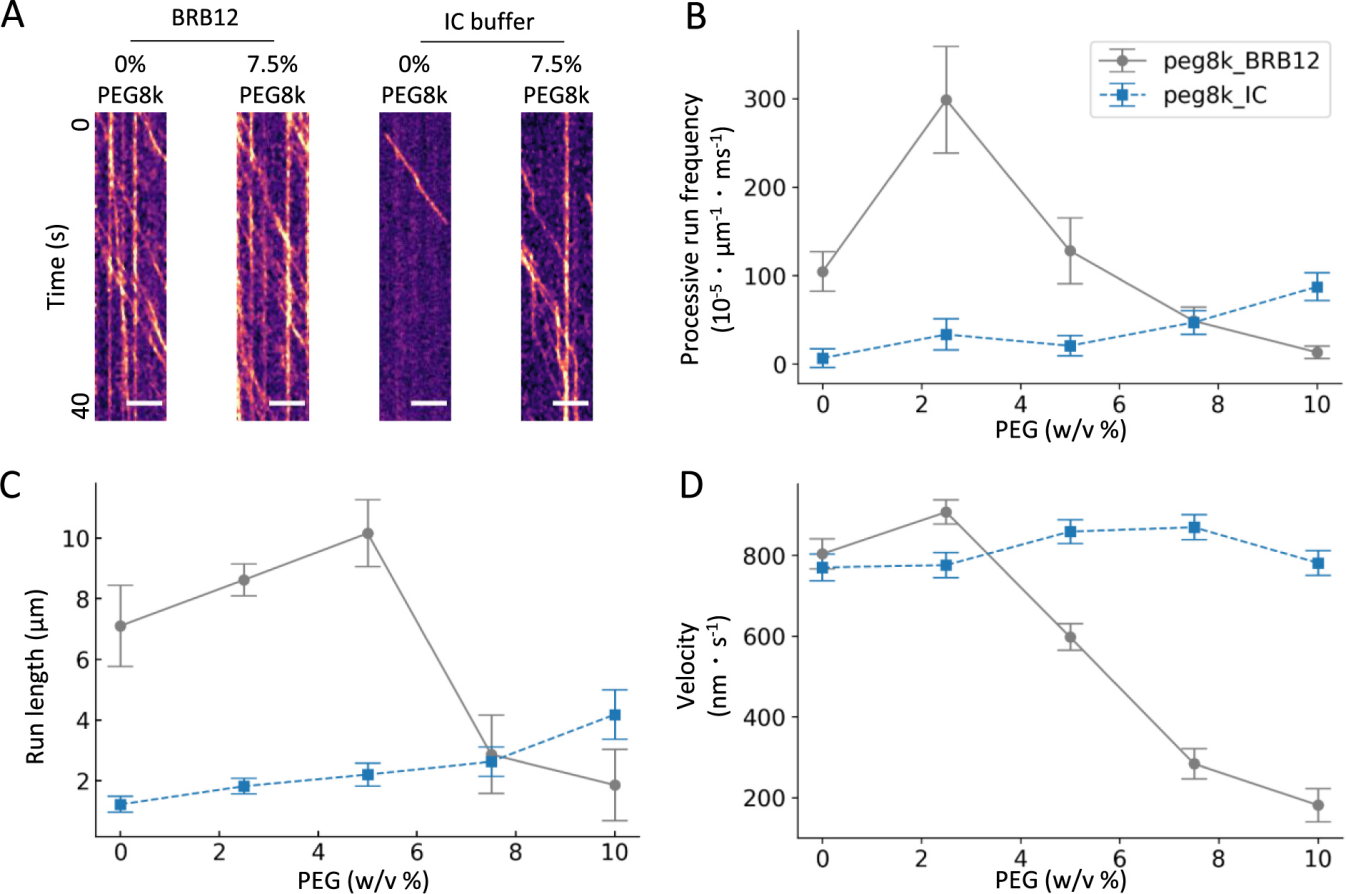
Single motility analysis of K430 in the presence of PEG8k under different buffer conditions (A) Representative kymographs of K430 movement in BRB12 and IC buffers containing PEG8k (0% or 7.5%). Scale bars represent 5 μm. (B, C, D) Quantitative analysis of K430 motility parameters under varying PEG8k concentrations: (B) processive run frequencies, (C) run lengths and (D) velocities. The gray line represents values in the BRB12 buffer, and the blue line represents those in the IC buffer. Error bars are SEM. The distribution of parameters and number of samples for each condition are described in Supplementary Materials (see Supplementary Materials Fig. S2, S3, and S4).

To evaluate how ionic composition and reduced diffusivity in cells influence K430 motility, we established an in vitro reconstitution system approximating intracellular conditions. The initial analysis focused on ionic strength effects by comparing the processive run frequency, run length and velocity of K430 between the low ionic strength BRB12 buffer and IC buffer, which mimics the cytosolic ionic composition (Fig. 3A). Although velocity remained comparable between conditions (Fig. 1C and 3D), the processive run frequency and run length were reduced significantly in the IC buffer compared with the BRB12 buffer (Fig. 3B and C), indicating suppressed processivity.

PEG was introduced to reconstitute intracellular crowding conditions. The average diameter of intracellular proteins is 3–6 nm (Milo and Phillips, 2015). PEG8k, which exhibits a similar hydrated diameter (Radius of gyration Rg = 3.7 nm) to the average size of intracellular proteins, was selected as a crowder (Holyst *et al.*, 2009). At ~8% (w/v) concentration, PEG8k produces a viscosity comparable to intracellular conditions (approximately 6.3 mPa·s) (González-Tello and Camacho, 1994). Considering that the size and aggregation state of PEG can vary with the salt concentration and that intracellular crowding and ionic strength fluctuate locally, the K430 motility assay was performed across a range of PEG8k concentrations to account for molecular crowding heterogeneity.

Adding PEG8k to the IC buffer increased the observed processive run frequency and run length of K430. In the BRB12 buffer, low concentrations of PEG8k initially enhanced these parameters, but they decreased sharply above a certain concentration threshold (Fig. 3B and C). Velocity measurements revealed a dramatic decrease in the BRB12 buffer with PEG8000 concentrations above 2.5%, whereas, remarkably, the velocity remained essentially unchanged with increasing PEG8k concentration in the IC buffer. Notably, under conditions most closely approximating the intracellular environment (IC with 8% PEG8k), K430 maintained its velocity while exhibiting enhanced processivity.

### Relationship between the molecular weight of the crowding agent and kinesin motility

**Fig. 4:**
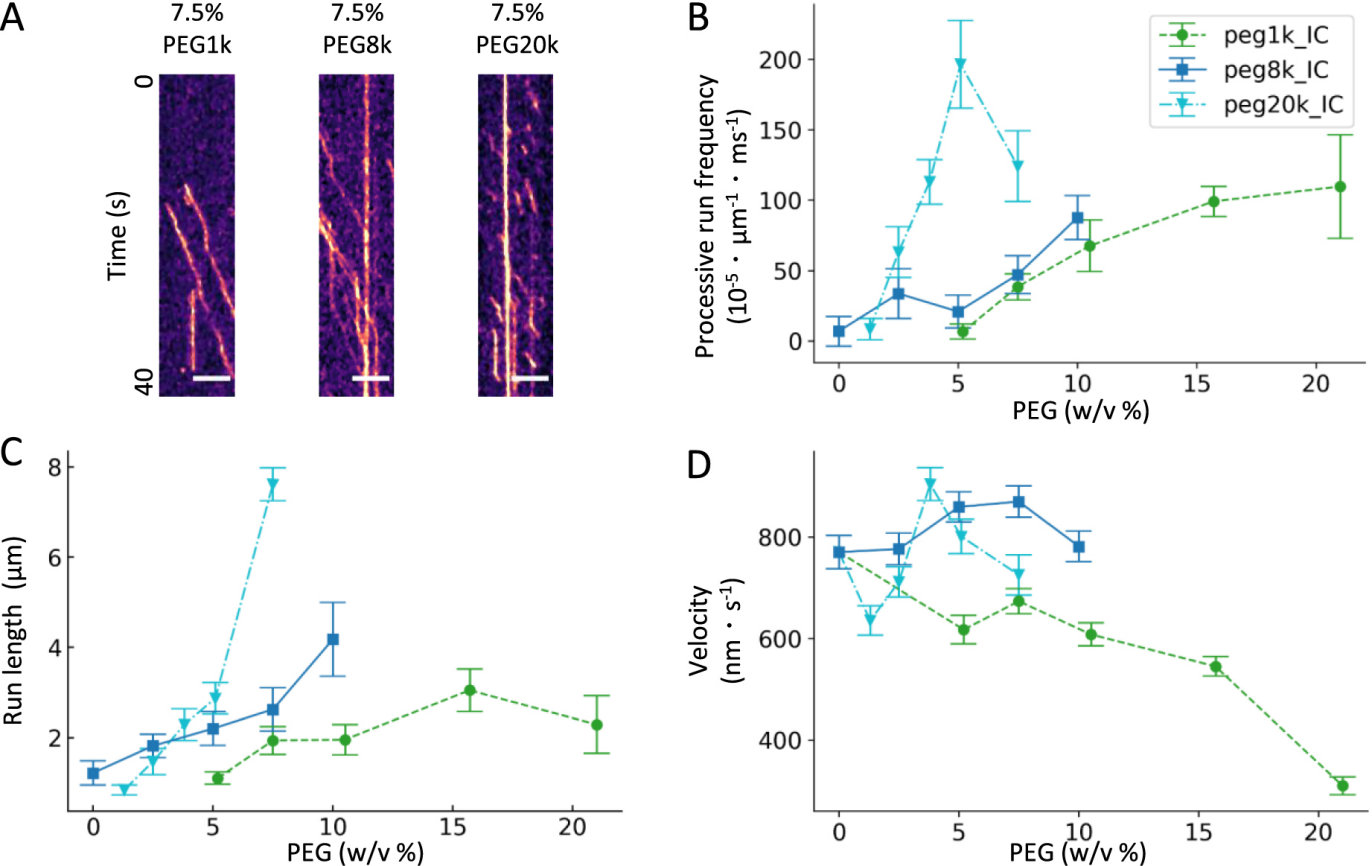
Single motility analysis of K430 motility in solutions containing different molecular weight PEGs (A) Representative kymographs of K430 motility in the IC buffer containing 7.5% PEG1k, 8k and 20k. Scale bars represent 5 μm. (B, C, D) Quantitative analysis of K430 motility parameters under varying PEG1k, 8k and 20k concentrations: (B) processive run frequencies, (C) run lengths and (D) velocities. The green line represents values with PEG1k, the blue line represents those with PEG8k, and the cyan line represents those with PEG20k. Error bars are SEM. The distribution of parameters and number of samples for each condition are described in Supplementary Materials (see Supplementary Materials Fig. S2, S3, and S4).

The results revealed that the velocity of K430 introduced into the cells was reduced, and this reduction was not reproduced in the in vitro reconstituted system when PEG8k was added to the IC buffer. It is possible that PEG8k has an optimal diffusivity and/or forms an optimal effective volume in which K430 can exist. To verify this, we performed a K430 kinetic analysis of the in vitro reconstituted system in which PEG of different molecular weights (PEG1k and 20k) was added to the IC buffer (Fig. 4A). The results showed that PEG20k was the most effective in increasing the processive run frequency and run length, whereas PEG1k was as efficient as or slightly less efficient than PEG8k (Fig. 4B and C). In terms of velocity, PEG8k was the most stable, whereas PEG20k showed a large variation among PEG20k concentrations, and PEG1k showed a consistent slowing trend. Focusing on PEG concentrations considered to be close to the intracellular viscosity [~10% (w/v) PEG1k, ~8% (w/v) PEG8k, and ~4% (w/v) PEG20k (González-Tello and Camacho, 1994; Sanyo Chemical Industries, 2021)], it was found that PEG8k and PEG20k maintained kinesin motility to a comparable degree, but in the presence of PEG1k, the kinesin velocity was decreased, indicating that the size of the crowder is important in maintaining kinesin velocity.

## Discussion

In this report, introduction of recombinant K430 into MEF cells revealed that the intracellular velocity of K430 was slower than that in the in vitro reconstituted system (Fig. 1C). Interestingly, this finding contrasts with previous reports (Hill *et al.*, 2004; Hirschberg *et al.*, 1998; Kural *et al.*, 2005; Naoi *et al.*, 2024; Ross, 2016) which often show faster intracellular transport than in vitro systems. This discrepancy may be attributed to using cargo binding motor proteins, such as beads and vesicles, or full-length motor proteins in prior studies. Motor proteins in a cargo-bound state are influenced by various motor activation factors, such as load-dependent activation (Coppin *et al.*, 1997), multimolecular cooperation through cargo binding (Furuta *et al.*, 2013; Tjioe *et al.*, 2019), and acceleration by cytosolic perturbations from actomyosin expansion and contraction (Ariga *et al.*, 2021). Full-length kinesins can be activated through interactions with regulatory proteins (Chiba *et al.*, 2022). In contrast, the K430 construct used herein, with its truncated tail domain, is less susceptible to these factors due to the absence of cargo binding and its smaller molecular size, resulting in no observed acceleration. Furthermore, the molecular crowding induced by intracellular macromolecules may significantly decelerate K430. In fact, K430 showed a decrease in velocity in the presence of PEG1k. Data for PEG8k and 20k cannot be shown because the viscosity was too high to observe movement of K430 at concentrations above those shown in Fig. 4 or because of the difficulty in making the flow chamber. However, if a local and transient intracellular situation were to occur in which the concentration of polymers with molecular weights greater than PEG8k reached a high concentration of more than 10%, kinesin motility would likely be inhibited there. Given the diverse sizes, shapes, and heterogeneous distributions of intracellular macromolecules (Dix and Verkman, 2008; Luby-Phelps, 2013), any of the conditions shown in Fig. 4 might be locally replicated in the intracellular environment.

Of particular significance regarding the effects on motility beyond velocity is the processivity of K430. The processive run frequency and run length of K430 were significantly reduced in the IC buffer simulating intracellular ionic strength. However, adding PEG8k, which simulates intracellular crowding, improved these parameters while preserving K430 velocity. This enhancement was consistent across PEGs of different molecular weights, suggesting that the depletion interaction from the exclusion volume effect (Asakura and Oosawa, 1958) reduces dissociation and promotes stable binding between K430 and microtubules.

The molecular mechanism underlying the differential effects of PEG8k on K430 motility remains to be elucidated, whereby K430 exhibits decreased velocity in BRB12 buffer but maintains its velocity in IC buffer at concentrations above 5% (Fig. 3). Our results from Fig. 2 demonstrate that the free diffusion of K430 is significantly restricted in IC buffer compared to BRB12 buffer in the presence of 5% PEG8k, suggesting that the diffusive properties of K430 in solution do not significantly affect its velocity. Since high ionic strength alone does not significantly alter K430 velocity (Fig. 1 and 3), we hypothesize that physicochemical changes of PEG8k under high ionic strength conditions contribute to the maintenance of K430 velocity. At PEG8k concentrations above 6.0%, molecular crowding effects become pronounced, resulting in molecular aggregation, compression, and overlap. In addition, under high ionic strength conditions, PEG molecules are known to dehydrate and adopt more compact conformations (Rubinson and Meuse, 2013; Saeki *et al.*, 1977). Considering these multiple contributing factors, further investigation focusing on the spatial organization and interactions among microtubules, K430, and PEG8k is warranted.

Overall, this study highlights the importance of macromolecules of appropriate size and concentration in maintaining the kinetic properties of kinesin in high ionic strength environments. This finding points to a novel mechanism for sustaining processivity, distinct from the previously known mechanisms involving complex formation and interactions with regulatory proteins. Future detailed analyses should shed light on the mechanisms responsible for motor protein motility, including the faster intracellular trafficking observed in other studies.

## Funding

This work was financially supported in part by JST SPRING (Grant Number JPMJSP2175) to M.S.

## Abbreviations

MEF: mouse embryonic fibroblast
IC buffer: intracellular-like buffer
PIPE: Photo-converted Intensity Profile Expansion
DDR2: Dendra2
LCI solution: Live Cell Imaging solution
PEG: polyethylene glycol

## Supplementary Material

Movie S1 Single-molecule imaging of K430 in a living cell. This is the movie of Fig. 1A. Cyan and magenta signals indicate transiently expressed tubulin-GFP and K430 introduced via liposome-based delivery, respectively. White arrow heads indicate representative unidirectional movement of K430. Scale bar: 2 μm.

Movie S2 Live cell imaging of K430 and FYVE, an early endosome marker. This is the movie of Fig. 1B. Magenta and green signals indicate liposome-delivered K430 and transiently expressed 2× FYVE-Venus, respectively. Yellow arrow heads indicate representative unidirectional movement of K430. The K430 movements were not associated with endosomal vesicles. Scale bar: 10 μm.
