# Supplementary material for: Impact of physiological ionic strength and crowding on kinesin-1 motility
Source: Cell Struct Funct. 2025 Jan 8;50(1):41–51. doi: 10.1247/csf.24074 (PMC12706510; doi:10.1247/csf.24074)
Supplement: Supplementary file 3 — Supplementary Materials [file csf_50_24074_3.pdf]

Supplementary Materials

Movie S1. Single-molecule imaging of K430 in a living cell.

This is the movie of Fig. 1A. Cyan and magenta signals indicate transiently expressed tubulin-GFP and K430 introduced via liposome-based delivery, respectively. White arrow heads indicate representative unidirectional movement of K430. Scale bar: 2 μm.

Movie S2. Live cell imaging of K430 and FYVE, an early endosome marker.

This is the movie of Fig. 1B. Magenta and green signals indicate liposome-delivered K430 and transiently expressed 2 × FYVE-Venus, respectively. Yellow arrow heads indicate representative unidirectional movement of K430. The K430 movements were not associated with endosomal vesicles. Scale bar: 10 μm.

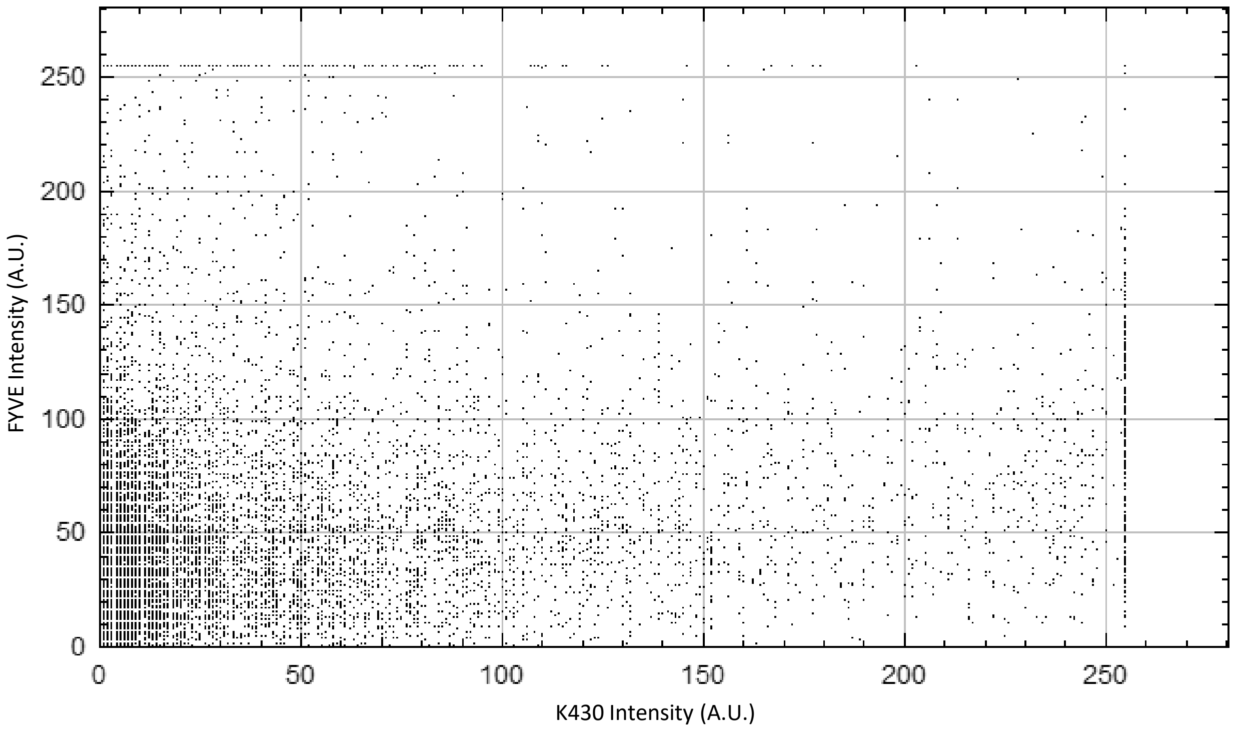

Fig. S1 Co-localization analysis of early endosomal markers FYVE and K430 in MEF cells

MEF cells expressing FYVE-GFP were treated with liposomes loaded with K430 using membrane fusion technology. The scatter plots show the spatial correlation between the fluorescence intensities of FYVE-GFP (*x*-axis) and K430 (*y*-axis), with each point corresponding to an individual pixel in the fluorescence microscope image(s). The lack of a correlation between the FYVE and K430 signals indicates that the K430-loaded liposomes were not incorporated into early endosomes and that liposomes remain independent of the early endosomal trafficking pathway.

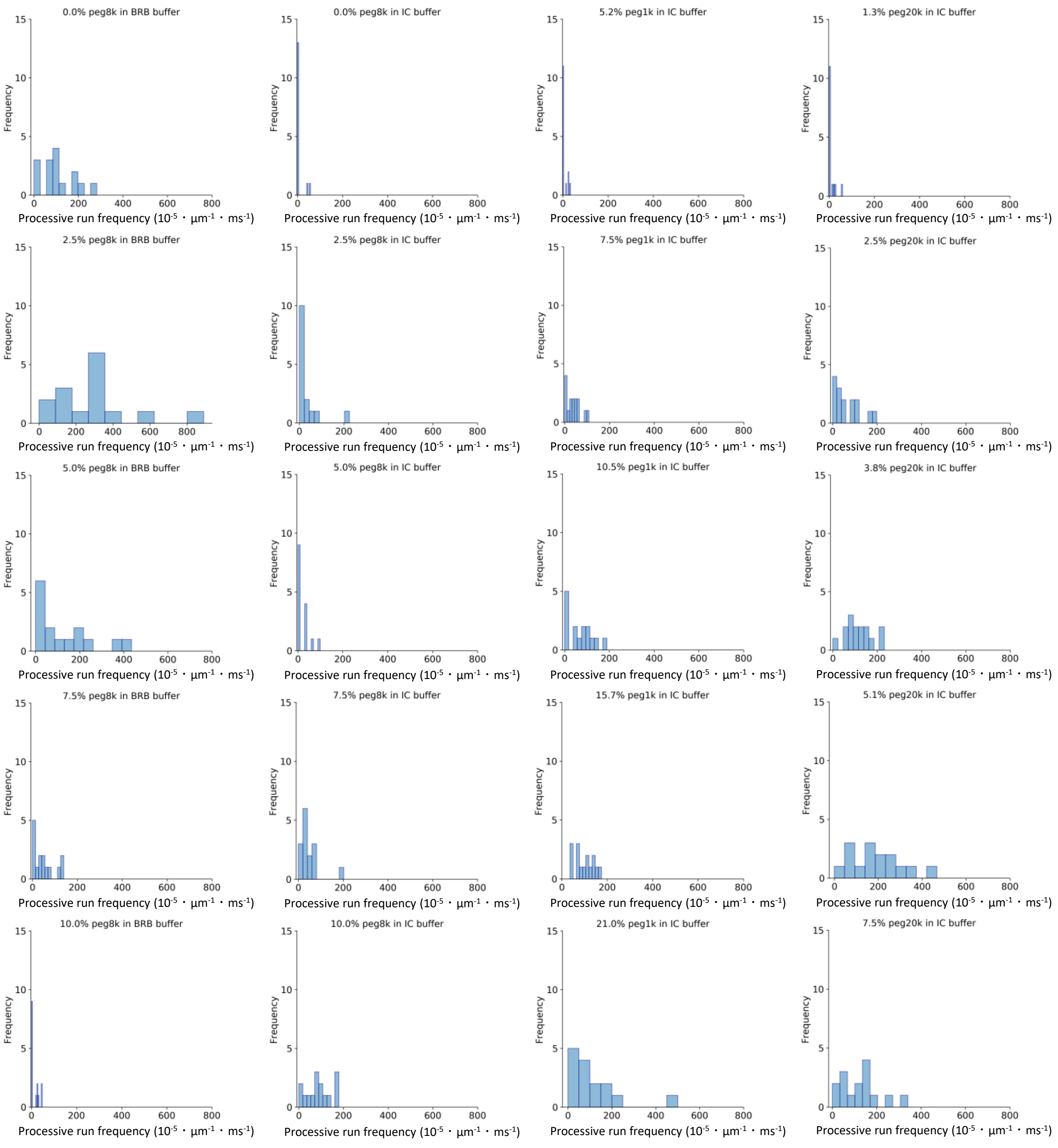

Fig. S2 Histogram of the processive run frequency under different experimental conditions

Frequencies represent the number of K430 motility events per microtubule; the *x*-axis represents the processive run frequency, and the *y*-axis represents the frequency of microtubules with the corresponding number of events. $n = 15$.

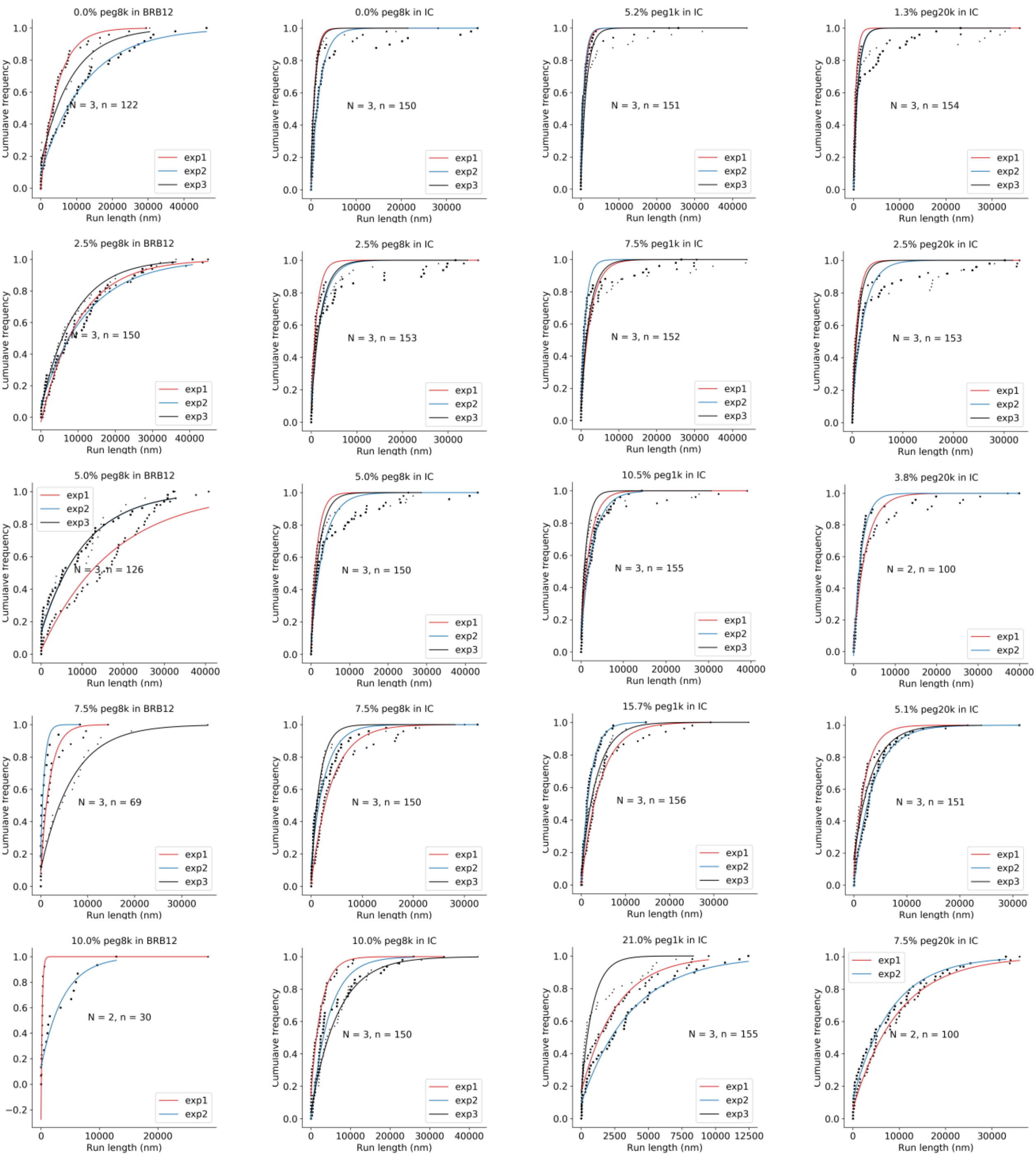

Fig. S3 Cumulative frequency distribution analysis of K430 run lengths under different conditions

The scatter plots show the experimental data, and the dashed lines indicate the fitted data. The distribution pattern reveals characteristic migration distances along the microtubules of the K430 molecule under each condition.

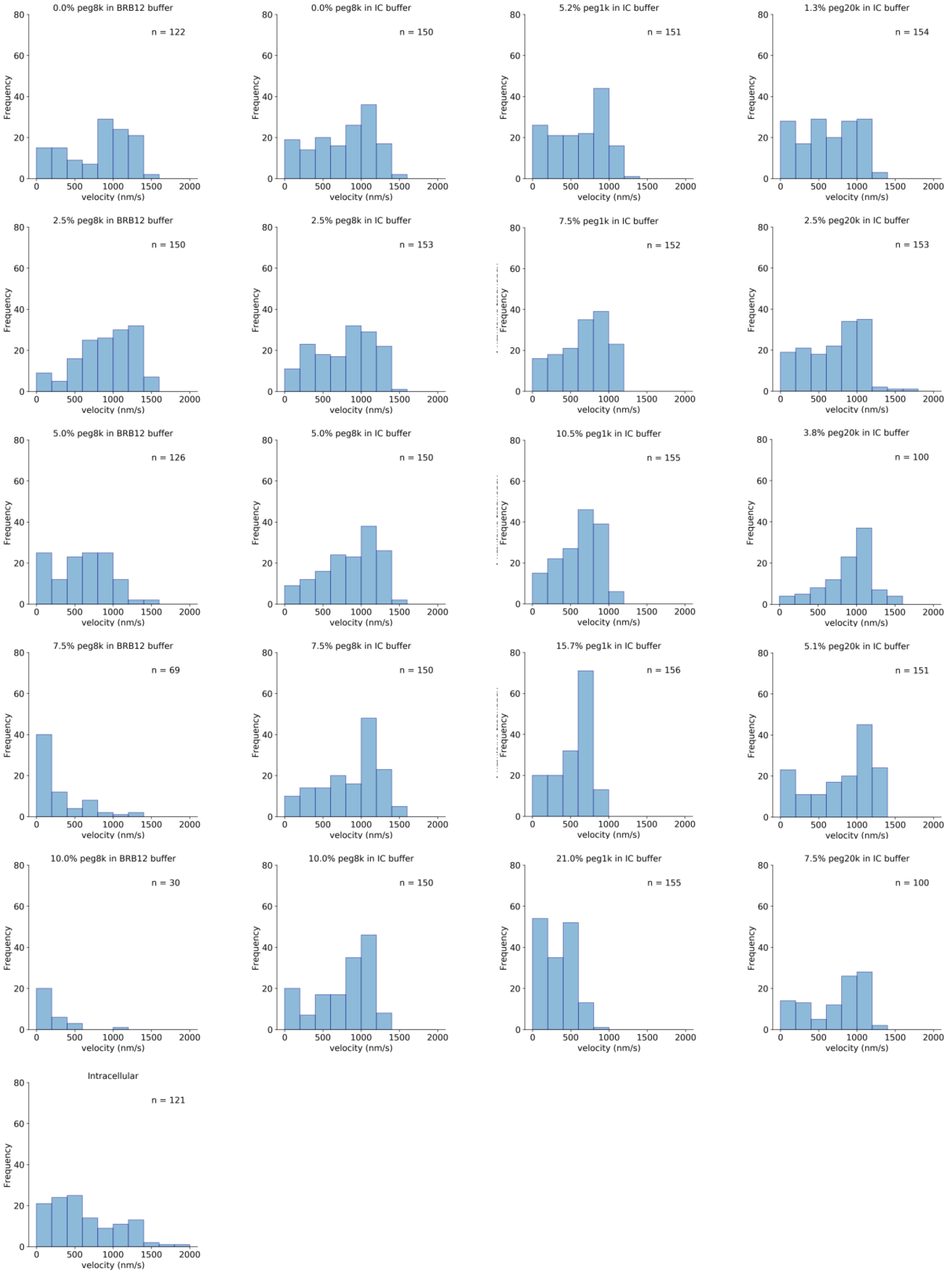

Fig. S4 Velocity distribution analysis of the K430 motor protein under different experimental conditions

Histograms showing the velocity distribution of K430 molecules measured under different conditions (buffer alone, PEG and all conditions combined). The x-axis represents the velocity (nm/s), and the y-axis shows the frequency of events.
